# Graphite-Mediated Microwave-Exfoliated Graphene Fluoride as Supercapacitor Electrodes

**DOI:** 10.3390/nano12111796

**Published:** 2022-05-24

**Authors:** Nicoloò Canever, Xianjue Chen, Mark Wojcik, Hui Zhang, Xinchen Dai, Marc Dubois, Thomas Nann

**Affiliations:** 1 School of Information and Physical Sciences, The University of Newcastle, Callaghan, NSW 2308, Australia; nicolo.canever@newcastle.edu.au; 2 School of Environmental and Life Sciences, The University of Newcastle, Callaghan, NSW 2308, Australia; 3 Allegro Energy Pty Ltd., Newcastle, NSW 2300, Australia; mark.wojcik@newcastle.edu.au; 4 School of Chemistry, The University of New South Wales, Sydney, NSW 2052, Australia; hui.zhang7@student.unsw.edu.au (H.Z.); xinchen.dai@unsw.edu.au (X.D.); 5 Institut de Chimie de Clermont-Ferrand (ICCF UME 6296), Université Clermont Auvergne, CNRS, 24 av. Blaise Pascal, F-63178 Clermont-Ferrand, France; marc.dubois@uca.fr

**Keywords:** graphene, graphene fluoride, microwave, exfoliation, supercapacitors

## Abstract

A graphite-mediated microwave-based strategy was used for solid-state exfoliation of graphite fluoride in a few seconds, followed by a simple yet efficient separation to obtain exfoliated materials based on the density difference between graphite and graphene fluoride in solvent. The microwave-exfoliated graphene fluoride was a few layers thick and electrically conductive. The electrochemical testing of pouch-cell supercapacitors assembled by using the exfoliated graphene fluoride electrodes and a novel microemulsion-based electrolyte showed reasonable performance with typical electrical double-layer capacitance behavior and good rate capability (gravimetric specific capacitance: 3.2 F g^−1^ at 500 mA g^−1^ and 3.1 F g^−1^ at 5000 mA g^−1^). The BET specific surface areas of the as-exfoliated graphene fluoride are ~60–80 m^2^ g^−1^, which could be increased by activation using this simple yet versatile microwave-based method for further improvements on the electrochemical performance.

## 1. Introduction

Graphite fluoride (GF) is a non-stoichiometric solid fluorocarbon compound that was first reported in 1934 [1,2]. It is a covalent-type graphite intercalation compound with a layered structure inherited from the starting graphite; depending on the fluorination conditions, two phases of GF have been obtained, i.e., stage-1 (CF)_n_, where fluorine atoms are bonded above and under graphene, and each carbon atom is covalently bonded to an F atom; and stage-2 (C_2_F)_n_, in which F atoms are intercalated into every second layer of graphite to establish an sp^3^ configuration with a ‘double-decked’ structure [3]. The (CF)_n_ type is commercially available, which has been used as a solid lubricant since the 1960s and as a cathode material in primary lithium batteries since 1973 [4,5].

Graphene fluoride (G–F) has been reported as a stable wide-bandgap two-dimensional material [6]. The strong and short C–F bond is responsible for the unique properties of G–F, including high thermal and chemical stability, hydrophobicity, and low surface energy and low friction coefficient between layers [7,8,9,10]. In addition to its being lightweight and having a high surface area, G–F has tunable electrical conductivity and electrochemical properties depending on the degree of fluorination and the nature of C–F bonding. It is a promising material for many energy-related applications. For example, it is considered to be one of the best cathode candidates for primary lithium batteries, given its abundant fluorine sites having a high affinity for lithium and a large specific surface area, facilitating the diffusion of lithium ions into the materials during the battery discharge process. Cheng et al. reported that adding 3 wt.% fluorinated graphene into an Li negative electrode led to a significant improvement in the rate capability and cycling life of Li–O_2_ cells [11]. Additionally, fluorinated carbon has been used as an efficient catalyst for electrochemical CO_2_ reduction, as the introduction of F could activate adjacent C atoms and promote catalytic activities [12]. Furthermore, a G–F-based supercapacitor provided a high pseudo-capacitance and improved the specific capacitance (227 F g^–1^), giving a power density of 50 kW kg^–1^ (at a current density of 50 A g^–1^) and good rate capability [13]. It was reported that the weakened covalent C–F bonds appear to be the key to achieve good performances in supercapacitors.

Two main routes for the synthesis of G–F are fluorination of graphene or its derivatives (e.g., graphene oxide) and exfoliation of GF [14]. The first route involves direct fluorination, such as exposure of graphene or its derivatives to a XeF_2_ or diluted F_2_ atmosphere [15,16]. Although this method can prepare high-quality fluorinated graphene with a precise control over the carbon-to-fluorine ratio and C–F bonding, the high cost and the special requirements in controlling the fluorination remain barriers for the scale-up of the material. In comparison, the ‘top-down’ exfoliation involving the use of commercially available GF as precursors is suitable for mass production, which includes liquid-phase exfoliation, ball milling, and thermal exfoliation [16,17,18,19,20,21,22]. In particular, the ‘thermal shock’ method has been used for the exfoliation of graphite oxide, where rapid heating induces sudden deoxygenation and the release of gases to overcome the van der Waals stacking of layers [23,24,25]. The thermal exfoliation was favored when GF contains residual fluorination catalysts (IF_5_, IF_6_^−^, and IF_7_). Although (C_2_F)_n_ and (CF)_n_ are prepared at a high temperature (350–650 °C) in pure F_2_ gas, fluorination of graphite with a high F/C atomic ratio can be achieved at room temperature, thanks to a gaseous catalytic mixture of F_2_, HF, and IF_5_ [26,27,28]. This material is usually denoted ‘room-temperature GF’. Moreover, the resulting sample exhibits a C–F bonding with weakened covalence because of the low fluorination temperature. The separation of graphene layers was enabled by the gaseous fluorinated species and the removal of IF_n_ species during the fast thermal decomposition.

Microwave-assisted processes have been used to produce or modify carbon materials and can also be combined with graphitic carbons acting as microwave absorbers to enhance the heating. We reported a ‘microwave-induced plasma’ method for the shock exfoliation of GF or graphite oxide [29,30]. The exfoliation occurs when the plasma interacts with GF through electron collision and/or charge exchange. The highly energetic species can also be obtained by graphite-mediated microwave reaction, where graphite acts as a ‘catalyst’ to convert electromagnetic energy into heat in a flash [31,32,33]. In this work, we found that graphite can enable the solid-state production of microwave-exfoliated graphene fluoride (MEGF) from room-temperature GF in few seconds, thanks to the weakened covalence of the C–F bonds and presence of IF_n_ residual catalyst. Furthermore, a simple yet effective strategy based on the density difference between graphite and MEGF was developed to separate MEGF. This method requires minimal energy input and does not involve the use of other chemicals. The as-prepared MEGF consists of crumpled, few-layers-thick electrically conductive nanosheets. Pouch cells were assembled by using the MEGF electrodes and a novel microemulsion-based electrolyte, which allowed the use of a high operating voltage, while still being mostly water-based. The electrochemical testing showed reasonable results, with typical electrical double-layer capacitance behavior and good rate capability. This simple yet versatile microwave-based method could allow further modulation of the defluorination of graphene fluoride and increasing the specific surface area by activation for improvements of the electrochemical performance.

## 2. Material and Methods

### 2.1. Preparation of MEGF

**Figure 1:**
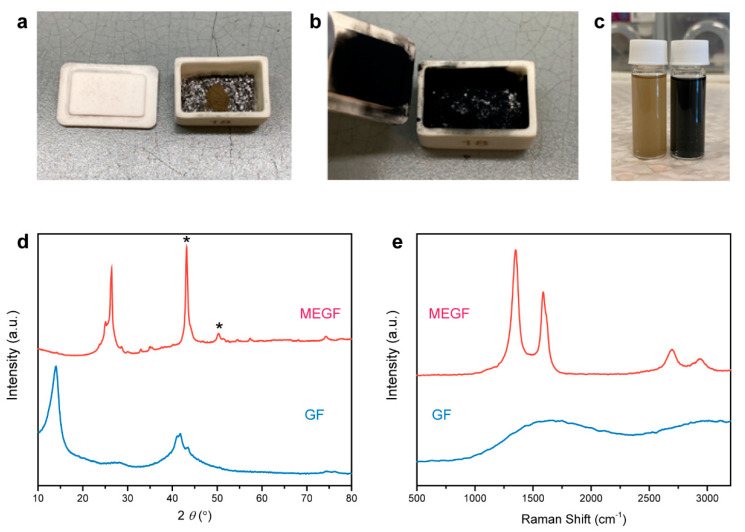
Photographs of GF before (**a**) and after (**b**) graphite-mediated, microwave-induced reaction that caused a significant expansion of GF and a color change from brown to black. The GF powder is placed on top of graphite flakes contained in an alumina crucible. (**c**) Photograph of GF (left) and MEGF (right) dispersed in ethanol showing brown and black colors, respectively. Powder XRD patterns (**d**) and Raman spectra (**e**) of GF and MEGF. The * peaks in (**d**) result from the XRD sample holder due to the low packing density of the fluffy MEGF sample.

GF with weakened C–F bonds was prepared by fluorinating graphite flakes in the presence of a gaseous mixture composed of a volatile fluoride (IF_5_, HF, and F_2_) at room temperature, and the synthesis procedure was described in previous studies [26,27,28]. The graphite flakes and commercial GF were purchased from Sigma-Aldrich (332461) and ACS Material (GT1FF012 and GT1F0020), respectively. The as-purchased graphite was first treated to remove smaller graphite flakes by using a 0.5 mm pore–size sieve. For a typical microwave treatment, 100 mg of the graphite with a lateral size > 0.5 mm was placed into an alumina crucible, and 20 mg of GF was added on top of the graphite without mixing (see Figure 1a). The crucible was then covered by an alumina lid and placed into a domestic microwave oven (Contempo, 900 W power). The reaction occurred immediately after turning on the microwave and was completed in a few seconds. Note that the experiments must be carried out in a fume hood to avoid possible contact of the released gases or MEGF powder. A secondary container (such as a larger glass beaker covered with a watch glass) for the crucible is recommended to limit spreading of the MEGF caused by the ‘violent fuming’. Further microwave treatment of the MEGF resulted in intense sparking or even damage of the crucible and glassware due to the flash heating. A proper amount of GF sample and an appropriately sized container with pressure-release pathway are suggested to avoid danger related to the sudden pressure increase and rapid release of gases. The crucible was left cooling naturally before removing from the microwave oven. The resulting MEGF and graphite flakes were added into ethanol. The supernatant suspension containing MEGF was transferred into another container after the graphite flakes settling down at the bottom. The MEGF solid was obtained by filtering the suspension on an anodic aluminum oxide membrane and dried in a vacuum oven at 40 °C overnight. The samples resulting from commercial GF flakes are denoted as MEGF-L (200–500 μm) and MEGF-S (1–10 μm).

### 2.2. Materials Characterization

Powder X-ray diffraction (XRD) patterns were collected on a Panalytical MPD and analyzed by using X’Pert HighScore Plus software. Raman spectra were acquired (Renishaw inVia 2 Raman Microscope) at room temperature with a 532 nm laser excitation. Scanning electron microscopy (SEM) images were recorded on a FEI Nova NanoSEM 450 FE-SEM under an accelerating voltage of 5 kV and a working distance of 5 mm. X-ray photoelectron spectroscopy (XPS) data were acquired by using an ESCALAB 250 Xi, Thermo Scientific. NMR experiments were carried out with Bruker Avance spectrometer, with working frequencies for ^19^F of 282.2 MHz. A Magic Angle Spinning (MAS) probe (Bruker) operating with 2.5 mm rotors was used. For MAS spectra, a simple sequence was performed with a single π/2 pulse length of 4.0 µs. ^19^F chemical shifts were externally referenced with respect to CFCl_3_. Transmission electron microscopy (TEM) specimens were prepared by drop casting an ethanol dispersion of material onto a 200-mesh holey carbon Cu grid (SPI Supplies, #2450-AB). Scanning transmission electron microscopy (STEM) elemental mapping was acquired by using a JEOL JEM-F200 Multi-Purpose FEG-S/TEM operating at an accelerating voltage of 200 kV. Image J and Gatan DigitalMicrograph were used for processing the TEM images. The N_2_ adsorption–desorption isotherms at −196 °C were recorded with a Micrometrics Tristar II surface area and porosity analyzer in order to measure the Brunauer–Emmett–Teller (BET) surface area and evidence the porosity type.

### 2.3. Electrode Preparation

The MEGF electrode slurries were manufactured by mixing as-prepared MEGF-L and MEGF-S, respectively, 83.33% by wt., 8.33% Ketjen Black (Lion Specialty Chemicals, Tokyo, Japan), and 8.33% PVDF as the binder (Sigma-Aldrich, St. Louis, MO, USA). N-methyl 2-pyrrolidone (NMP) (Sigma-Aldrich) was added until the mixture reached a desired viscosity. The slurries were magnetically stirred overnight to ensure a homogeneous mixture. The slurries were then ‘doctor blade’ coated onto a graphite foil (120 mm thick, Ceramaterials, Dingmans Ferry, PA, USA) and dried in a vacuum oven for 12 h at 180 °C to remove the NMP. The areal loadings of the two samples were 3.39 mg cm^−2^ (MEGF-S) and 3.18 mg cm^−2^ (MEGF-L). Similarly, an activated carbon electrode slurry was manufactured by replacing the MEGF above with 83.33 wt.% activated carbon (Sigma-Aldrich—activated charcoal DARCO^®^, −100 mesh particle size, powder). The areal loading was 4.08 mg cm^−2^.

### 2.4. Electrolyte Preparation

The microemulsion electrolyte consisted of a mixture of 60 wt.% distilled water, 8.33 wt.% sodium dodecyl sulfate (SDS) (Tokyo Chemical Industry, Tokyo, Japan), 16.67 wt.% n-butanol (Sigma-Aldrich), and 15 wt.% cyclohexane (Sigma-Aldrich). A clear homogeneous solution was observed which confirmed the formation of the microemulsion. Then 0.1 M NaCl (Sigma-Aldrich) was added to the mixture. This electrolyte was used for all supercapacitor experiments.

### 2.5. Supercapacitor Cell Assembly

Glass microfiber (Grade GF/D, Whatman) was used as the separator between the electrodes. The assembled cells were then inserted into a laminated aluminum film pouch (from inside to out: polypropylene/aluminum foil/polyamide). Each separator was soaked with approximately 0.9 mL of microemulsion. The pouch was then sealed under vacuum. Supercapacitor cell assembly was performed on a lab bench under ambient conditions.

### 2.6. Electrochemical Testing

Cyclic voltammetry (CV), electrochemical impedance spectroscopy (EIS), and galvanostatic charge–discharge (GCDC) at increasing upper cutoff voltage experiments were carried out by using a Metrohm Autolab (Multi Autolab M204). EIS was performed over a frequency range of 0.1 Hz to 10 kHz, using a perturbation amplitude of 10 mV. GCDC at increasing scan rate, voltage hold, and leakage current testing was performed on a Neware CT-4008T-5V12A battery analyzer system. All testing used the cathode as the working electrode and anode as the reference and counter electrode. Gravimetric specific capacitances were calculated by dividing the capacitance values obtained via galvanostatic cycling by the combined mass of anode and cathode. Leakage currents were measured by charging the devices to 2.5 V and holding such voltage for at least five hours. The current value at the 5 h mark (divided by the mass of the electrodes) is the resulting leakage value current.

## 3. Results and Discussion

In a typical experiment, an alumina crucible containing GF powder (20 mg) on top of a layer of graphite flakes (100 mg) was placed in a domestic microwave oven for the reactions which occurred and was completed within a few seconds. A large volume expansion of the GF powder was observed, accompanied by ‘violent fuming’ yielding a black and fluffy MEGF powder covering surfaces inside the crucible (Figure 1a,b). The generation of sparks appears to be a prerequisite for the occurrence of the reaction. Graphite is an excellent microwave absorber that is capable of converting electromagnetic energy into heat under microwave irradiation [34,35,36]. The de-localized π-electrons in graphite can respond to the oscillating electromagnetic field, resulting in a large potential accumulation on surfaces. When two graphite flakes are next to each other, the potential difference allows electrons with high kinetic energy to pass from one graphite to another, while they ionize the local air, yielding microscopic plasma in the form of sparking [37]. Note that no exfoliation occurred in the absence of graphite or when mixing GF with graphite. Sparking can be generated only when the graphite flakes are sufficiently close to each other and are not ‘diluted’ by GF, a poor microwave absorber [27]. The sparking induced rapid exothermic decomposition of GF, providing additional driving force for a self-propagating reaction.

The separation of graphite flakes and MEGF is required, as they are well mixed after the reaction, as shown in Figure 1b. Dispersing the mixture into ethanol allows an effective separation because large graphite flakes quickly sink to the bottom, while MEGF can be temporarily stabilized in the supernatant and separated by filtration (Figure 1c). To obtain pure MEGF, a key step is to remove small flakes/fragments in the graphite ‘catalyst’ by sieving prior to the reaction, as they are otherwise difficult to separate from the mixture by using the same strategy. We note that this separation route based on density difference is potentially applicable to other graphite-mediated reactions.

The powder XRD pattern of GF shows a peak at a 2*θ* value of 14.0° (interlayer spacing d_i_ in between 0.60 and 0.66 nm) should be assigned as 001 reflection. A peak around 21° is expected for room-temperature GF and is related to the catalyst residual species intercalated between the graphite layers (002 reflection of the stage-one IF_5_-GIC [14]); its low intensity suggests the low content of iodine species (Figure 1d). The (001) peak disappeared after microwave treatment, while the (002) peak from the recovered graphitic structure appeared. Raman D, G, and 2D bands were observed for MEGF, while GF showed a quenched Raman activity due to its wide bandgap (Figure 1e). The G band is an in-plane vibrational mode involving the sp^2^-bonded carbon in graphene, which is typically used for determining the degree of graphitization and the number of layers present in graphene samples [38]. The G band at 1587 cm^–1^ is indicative of the presence of repaired sp^2^-bonded carbon in MEGF. This recovery of the graphitic structure caused by the cleavage of C–F bonds has been reported, which is different from the case of graphene oxide where divalent oxygen tends to form C=O bonds and breaks C–C bonds during deoxygenation [39]. The D band represents a ‘breathing mode’ from sp^2^ carbon rings in graphene, which becomes active when the rings are adjacent to edges or defects. The intensity of the D band is indicative of the level of defects in the sample. Thus, the D band at 1351 cm^–1^ is associated with the residual F chemisorption on graphene and structural disorder/defects generated by exfoliation and decomposition that also led to the fragmentation of MEGF and increase of the number of edge sites. Consequently, a relatively large intensity ratio of D and G bands (*I*_D_/*I*_G_ = 1.5) was observed.

**Figure 2:**
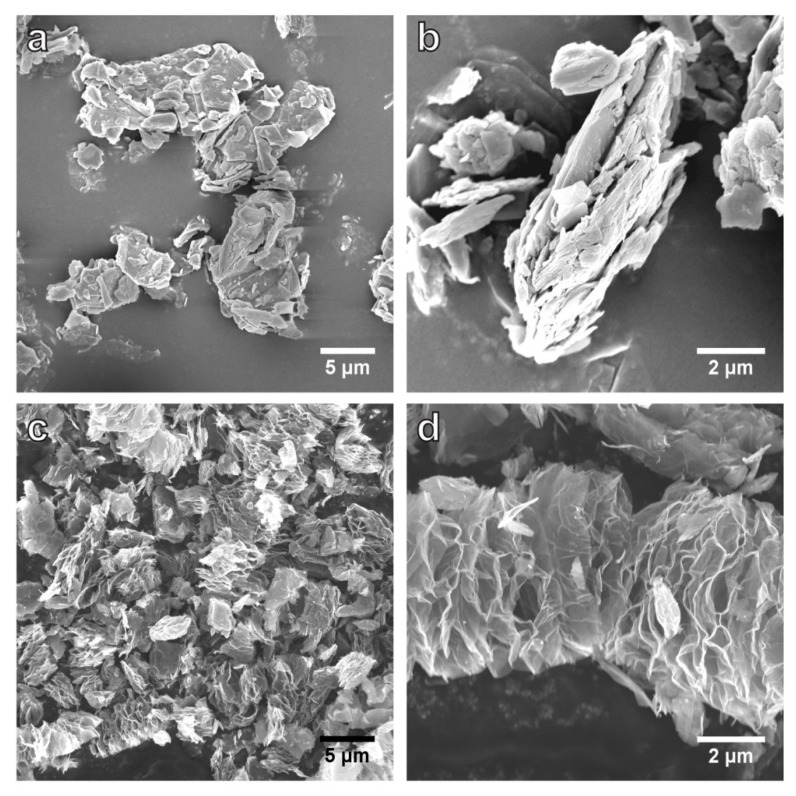
SEM images of GF (**a**,**b**) and MEGF (**c**,**d**). The MEGF shows a heavily expanded structure due to the rapid release of evolved gases expanding the layers along the c-axis direction after the graphite-mediated reaction.

SEM images (Figure 2) show the GF flakes that are 2–20 µm in lateral size, with a layered structure. MEGF features a ‘worm-like’ morphology with expanded, undulating layers that are connected to yield a porous structure. The pores between the layers are generated by the sudden escape of the evolved gas species, e.g., CF_4_, C_2_F_4_, and C_2_F_6_, during the reaction. SEM imaging induced strong surface charging on GF (without a thin layer of Pt coating) but not on bare MEGF, suggesting that electrons cannot easily move through the non-conductive GF, while MEGF is electrically conductive. Additionally, GF or graphite is absent in the MEGF sample, and this is indicative of a complete decomposition of GF and separation of MEGF from the graphite flakes.

**Figure 3:**
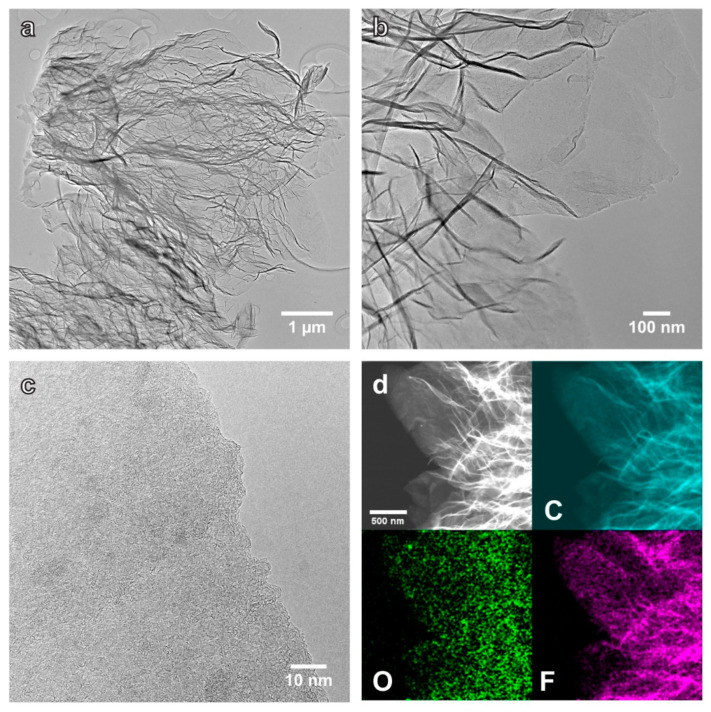
(**a**,**b**) Bright-field TEM images of the MEGF platelets. (**c**) TEM image over an edge area of a thin MEGF platelet, showing a heavily distorted structure. (**d**) STEM-HAADF image and elemental maps of C, O, and F on the MEGF platelets.

Typical TEM images (Figure 3) of MEGF sheets (first dispersed in ethanol and dried in air) have many wrinkled and folded regions caused by the exfoliation and the presence of structural disorder/defects. A magnified image (Figure 3c) over an edge area of a MEGF platelet shows a heavily distorted structure with irregularly stacked layers. Scanning transmission electron microscopy (STEM) high-angle annular dark-field (HAADF) image and element maps of MEGF indicate the uniform presence of C, O, and F in the platelets.

**Figure 4:**
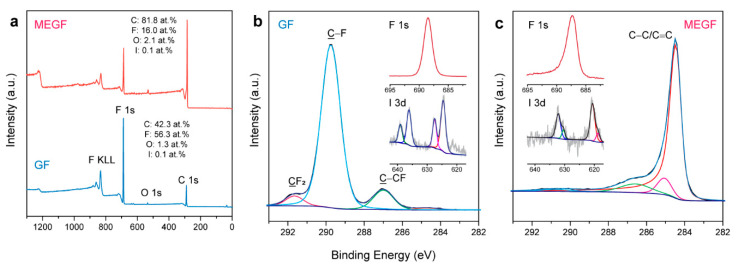
XPS surveys (**a**) and C 1s spectra (**b**,**c**) of GF and MEGF. The insets show the F 1s and I 3d spectra. The colored lines show the curve fitting components.

Figure 4a shows the XPS survey spectra of GF and a typical batch of MEGF, where their compositions can be quantified. GF is composed of C (42.3 at.%), F (56.3 at.%), O (1.3 at.%), and I (0.1 at.%). The high F/C ratio (1.3) is related to the presence of IF_5_, IF_6_^−^, IF_7_, and HF_2_^−^ intercalated species [31,32,33] and CF_2_ and CF_3_ groups in the periphery of amorphous regions and/or edge sites [40]. The reaction caused a significant defluorination with a dramatic decrease of the F/C ratio to 0.2, although F (16.0 at.%) is still present in MEGF. There is a slight increase of O content to 2.1 at.%, as a result of oxidation in air. The defluorination led to a strong chemical shift in the C 1s binding energy (Figure 4b,c). For GF, an intense peak with a binding energy of 289.8 eV corresponds to the sp^3^-bonded C atoms in the C–F bonds. The peak at 287.0 eV is associated with the non-fluorinated C atoms linked to F-bound carbons (C–CF species). The peak at 291.7 eV corresponds to the C atoms in the CF_2_ groups in the amorphous regions and/or edge sites. For MEGF, the restoration of graphitic structure is evident from the intense peak at 284.5 eV related to the C atoms in the sp^2^-bonded configurations. The low intensity of the peaks associated with the C–F bonds suggests significant defluorination.

A characteristic peak was observed at 688.5 eV in the F 1s spectrum of GF, corresponding to the covalent F species present in the highly fluorinated carbon. For MEGF, a slight shift of ~1.2 eV (687.3 eV) is evident, implying a change in the chemical environment for the F atoms after defluorination, i.e., the transformation into a more weakened covalence of C–F bonds because more non-fluorinated sp^2^ C is present in the neighboring [41]. This is evident in the ^19^F NMR spectrum (Figure S1) showing the coexistence of both covalent C–F and weakened covalence due to hyperconjugation in MEGF [21,42]. The line corresponding to the CF_2_ groups can be observed. The absence of lines at 0–50 ppm suggests that the IF_n_ intercalated species were largely removed. The XPS I 3d spectrum of MEGF (Figure 4c inset) featured two fitted peaks at 618.7 and 620.7 eV in the I 3d_5/2_ component, indicating the presence of I_3_^−^ and I_5_^−^ anions [43], which likely remain in the MEGF as dopants.

The present microwave exfoliation method was extrapolated to commercial covalent-type GF with two different flake sizes, MEGF-L (200–500 μm) and MEGF-S (1–10 μm). The products show similarities in terms of the exfoliated morphology, as shown in the SEM images (Supplementary Figure S2). The exfoliation of the two samples after microwave treatment is evident from the disappearance of the XRD (001) peaks and recovery of the (002) peaks (Supplementary Figure S3). Note that a strong (002) peak is present in GF-L, indicating incomplete fluorination, likely due to the relatively large flake size of the pristine graphite. XPS spectra (Supplementary Figure S4) show F/C ratios of 0.06 and 0.18 for MEGF-L and MEGF-S, respectively. The MEGF-L features relatively low F and O contents of 5.6 and 1.7 at.%, respectively, which might be attributed to the incomplete fluorination, as evidenced by XRD. In comparison, MEGF-S shows F and O contents of 14.1 and 4.9 at.%, respectively. XPS C 1s spectra of MEGF-L and MEGF-S show chemical shifts before and after exfoliation, suggesting the presence of C–F bonding with weakened covalence. The microwave-based strategy allows ultrafast simultaneous defluorination and exfoliation of GF regardless of their nature, whether semi-covalent (room temperature GF) or covalent (commercial GF). The two types of MEGF prepared from commercial products were investigated in electrochemical tests, as discussed below.

**Electrochemical performances:** Powder composed of highly exfoliated graphene platelets can be a promising electrode material for supercapacitors due to its high surface area and good electrical conductivity. The rapid exfoliation of GF by graphite-mediated microwave heating described here may provide a simple and scalable route to prepare graphene-based materials for electrical energy storage. Here, we prepared MEGF-L and MEGF-S as electrodes in pouch-cell supercapacitors. A novel microemulsion-based electrolyte was used in the devices: this electrolyte, while mostly composed of water, with a small quantity of an organic solvent serving as an ‘oil’ phase, can allow a significant expansion of the working voltage window of the electrochemical devices. This is due to the formation of an ‘oil-rich’ layer on the surface of the electrode that prevents water molecules from encountering it, thus inhibiting the irreversible water-splitting reaction [44]. The formation of this layer is favored by the hydrophobic character of MEGF thanks to the presence of residual fluorine atoms. Additionally, this electrolyte achieves this extension of voltage window without relying on large quantities of expensive organic solvents, or using high concentration of expensive salts, such as in the case of water-in-salt electrolytes. Finally, the predominance of water in the electrolyte compositions renders the use of a glovebox redundant, and the cells can thus be assembled in ambient conditions.

**Figure 5:**
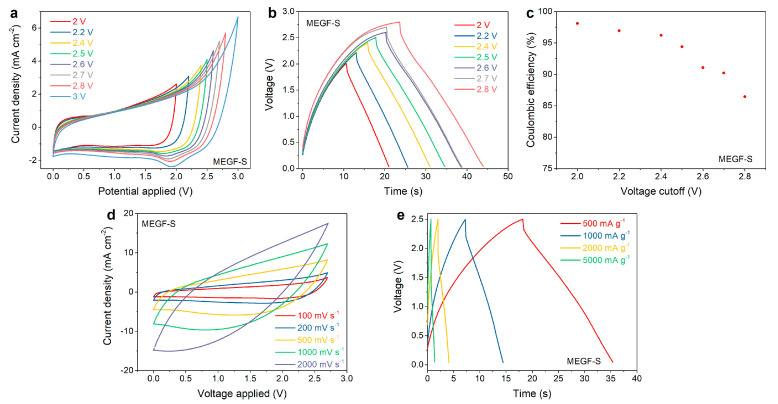
Electrochemical data for MEGF-S: cyclic voltammograms with increasing upper vertex voltages (**a**), Galvanostatic charge–discharge curves with increasing upper voltage cut-off (**b**) and relative Coulombic efficiencies (**c**), cyclic voltammograms at increasing scan rates (**d**), and galvanostatic charge–discharge curves at increasing current densities (**e**).

It can be seen from Figure 5a that the cyclic voltammograms acquired for the supercapacitors constructed by using MEGF-S materials show no sign of water splitting and highly reversible capacitive behavior up to voltages as high as 2.7–2.8 V. It can also be seen that, when using higher upper voltage vertices, a reduction peak becomes increasingly evident: this is likely due to the presence of surface defects and edge sites in the material, as confirmed by the prominent D band in the Raman spectra. These sites can promote redox reactions, causing some pseudo-capacitive behavior. Correspondingly, the galvanostatic charge–discharge curves acquired at 500 mA g^−1^ (Figure 5b) show good triangular-shaped voltage trends, retaining high (>94%) Coulombic efficiency at voltages up to 2.5 V (Figure 5c). This voltage is significantly higher than what can normally be obtained by using water-based electrolytes, approaching the performance of organic electrolytes. The rate capability of MEGF-S was also tested by performing cyclic voltammetry and galvanostatic charge–discharge experiments at increasing scan rates and current densities. It can be seen from Figure 5d that, with the increase of the scan rate, the device shows a gradually increasing twist in the shape of its hysteresis; this is also reflected by the gradual increase of IR drop in Figure 5e with the increasing of the applied current density.

**Figure 6:**
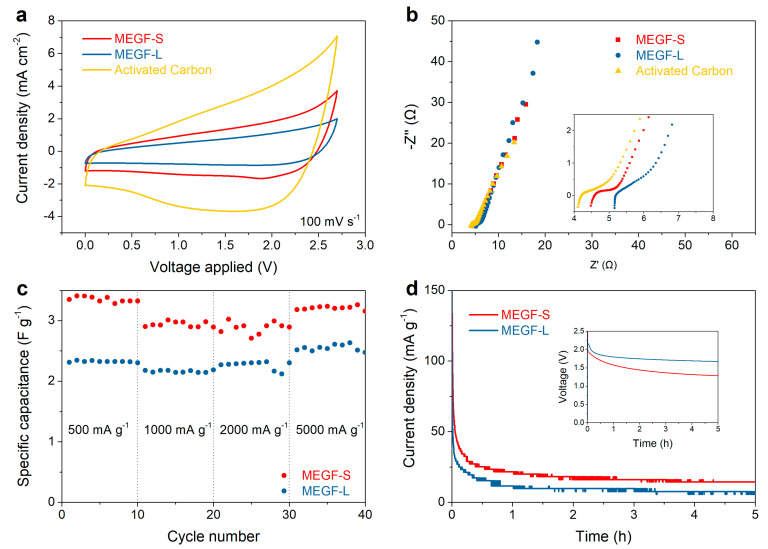
Comparison of the electrochemical performance for MEGF-S, MEGF-L, and activated carbon: cyclic voltammograms acquired at 100 mV s^−1^ (**a**), Nyquist plots (**b**), specific discharge capacitance at increasing current intensities (**c**), and voltage hold tests at 2.5 V (inset: self-discharge curves after charging the devices to 2.5 V) (**d**).

The electrochemical tests were repeated for both MEGF-L and MEGF-S, and their performance was also compared to a commercially available activated carbon material to test how the material compares to commonly used supercapacitor electrode materials. This material has shown promising results in preliminary tests and was therefore picked as a reference. Figure 6a shows that, while both MEGF-L and MEGF-S show typical dual-layer capacitive behavior, as shown by the rectangular shape of their respective voltammograms, MEGF-S shows notably higher current densities, translating into a higher specific capacitance. A small hump in the cathodic current is also present in the case of MEGF-S, likely indicating the presence of pseudocapacitive behavior. This could be caused by the redox reaction of functional groups and defects present in the material. It is also worth noting, however, that both materials are performing worse than the more conventionally used activated carbon. N_2_ adsorption–desorption isotherms (Supplementary Figure S5) indicate lower adsorbed volume for MEGF-S and MEGF-L than for the case of AC, especially in the micropore range. Moreover, the BET surface areas of 805, 76, and 63 m^2^ g^–1^ for activated carbon, MEGF-L, and MEGF-S, respectively. The performance gap is most likely due to the much higher specific surface area of the commercial activated carbon compared to that of MEGF; another possible explanation is the high atomic percentage of residual fluorine or defect in the MEGF which could be hampering their electrical conductivities. Future work could explore the effect on electrochemical performance by varying degrees of defluorination on MEGF and/or increasing the specific surface area through activation.

Electrochemical impedance spectroscopy (EIS) was also performed on the two samples. It can be seen from Figure 6b that the low-frequency region of the Nyquist plot shows a near-vertical trend, indicating the good capacitive behavior of the materials. The size of the semicircle in the high-frequency region, corresponding to the charge transfer resistance, is of a similar magnitude for all tested materials. While the slope of the diffusion region and the semicircle relative to the charge transfer resistance are virtually identical for MEGF-S and MEGF-L, the trend observed in the cyclic voltammetry data is confirmed in the in-series resistance, approximated as the intercept of the data with the x-axis: MEGF-S shows a lower value than MEGF-L, indicating lower in-series resistance and higher conductivity. This could be attributed to MEGF-S forming a better electrical contact with the current collector. Activated carbon, however, shows the lowest in-series resistance of the materials tested. The galvanostatic cycling data at varying current densities shows good rate capabilities for both MEGF-L and MEGF-S, as virtually ideal capacity retention is observed at currents up to 5000 mA g^−1^. The voltage hold and self-discharge tests were also performed on the devices: Figure 6d shows that, after charging the cells and holding the voltage to 2.5 V, a lower leakage current of 7.5 mA g^−1^ was observed for MEGF-L, compared to a value of 14.3 mA g^−1^ for MEGF-S. This is consistent with the previous observations of lower in-series resistance for MEGF-S, which translates to a faster rate of self-discharge for devices, using the material as electrode.

## 4. Conclusions

Graphite flakes have been used as ‘catalyst’ for solid-state shock exfoliation of graphite fluoride in conventional microwave oven within few seconds. The density difference between graphite and the microwave-exfoliated graphene fluoride (MEGF) allowed an efficient separation after reaction. Regardless of the nature of graphite fluoride and its C–F bonding, this simple yet efficient process has provided a scalable method to generate MEGF that could be used, among other applications, as an electrode material in energy-storage devices, such as supercapacitors. The electrochemical testing showed typical electrical double-layer capacitance behavior and reasonable rate capability. Future improvements on the electrochemical performance are possible through controlling the degree of defluorination of MEGF (e.g., through thermal annealing) and/or increasing its specific surface area by activation.

## Supplementary Materials

The following supporting information can be downloaded at: https://www.mdpi.com/article/10.3390/nano12111796/s1, Figure S1: ^19^F MAS (20 kHz) spectrum of MEGF; Figure S2: SEM images of MEGF-L and MEGF-S; Figure S3: XRD patterns of GF-L and MEGF-L and GF-S and MEGF-S; Figure S4: XPS surveys, C 1s and F 1s spectra of GF-L, GF-S, MEGF-L, and MEGF-S; Figure S5: N_2_ adsorption-desorption isotherms of activated carbon, MEGF-L, and MEGF-S.
