# Supplementary material for: Graphite-Mediated Microwave-Exfoliated Graphene Fluoride as Supercapacitor Electrodes
Source: Nanomaterials (Basel). 2022 May 24;12(11):1796. doi: 10.3390/nano12111796 (PMC9182021; doi:10.3390/nano12111796)
Supplement: Supplementary file 1 [file nanomaterials-12-01796-s001.zip › nanomaterials-1704778 supplementary proof.pdf]

nanomaterials

MDPI

Supplementary Materials

Graphite-Mediated Microwave-Exfoliated Graphene Fluoride as Supercapacitor Electrodes

Nicolò Canever [1], Xianjue Chen [2,*], Mark Wojcik [3], Hui Zhang [4], Xinchen Dai [4], Marc Dubois [5] and Thomas Nann [1,3,*]

[1] School of Information and Physical Sciences, The University of Newcastle, Callaghan, NSW 2308, Australia; nicolo.canever@newcastle.edu.au
[2] School of Environmental and Life Sciences, The University of Newcastle, Callaghan, NSW 2308, Australia
[3] Allegro Energy Pty Ltd., Newcastle, NSW 2300, Australia; mark.wojcik@newcastle.edu.au
[4] School of Chemistry, The University of New South Wales, Sydney, NSW 2052, Australia; hui.zhang7@student.unsw.edu.au (H.Z.); xinchen.dai@unsw.edu.au (X.D.)
[5] Institut de Chimie de Clermont-Ferrand (ICCF UME 6296), Université Clermont Auvergne, CNRS, 24 av. Blaise Pascal, F-63178 Clermont-Ferrand, France; marc.dubois@uca.fr
* Correspondence: sam.chen@newcastle.edu.au (X.C.); thomas.nann@newcastle.edu.au (T.N.)

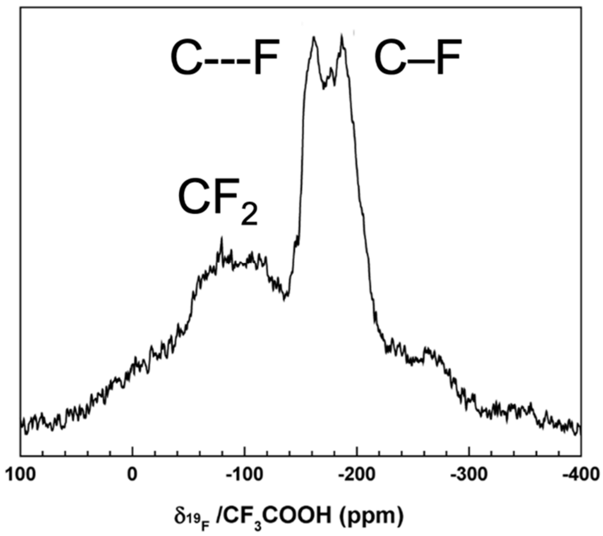

Figure S1. ^{19}F MAS (20 kHz) spectrum of MEGF.

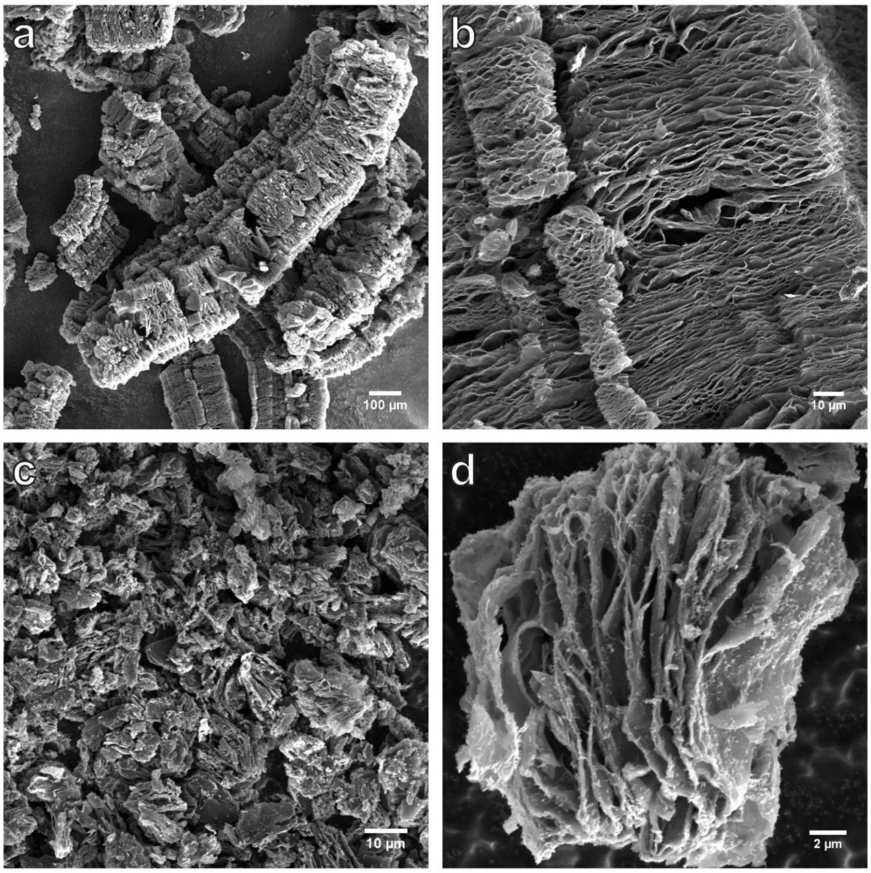

Figure S2. SEM images of MEGF-L (**a**,**b**) and MEGF-S (**c**,**d**).

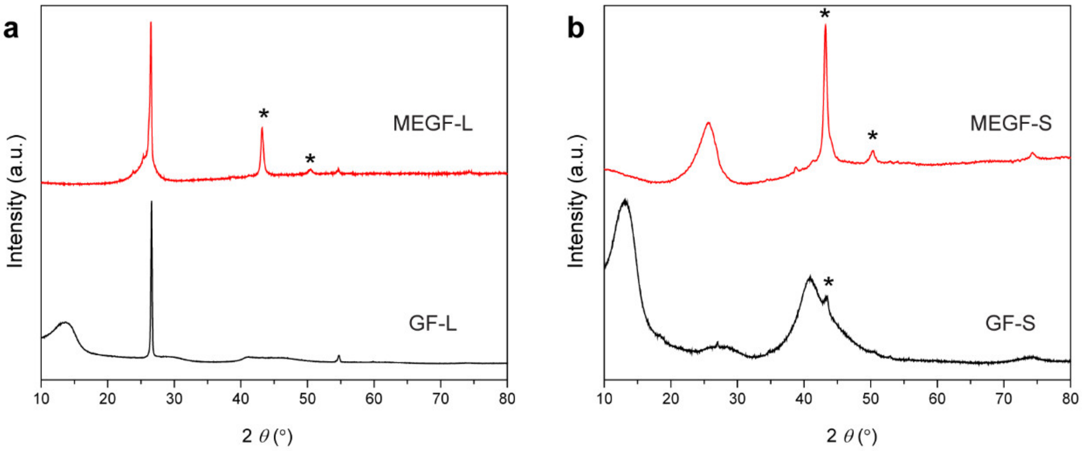

Figure S3. XRD patterns of (**a**) GF-L and MEGF-L and (**b**) GF-S and MEGF-S. The * peaks result from the XRD sample holder.

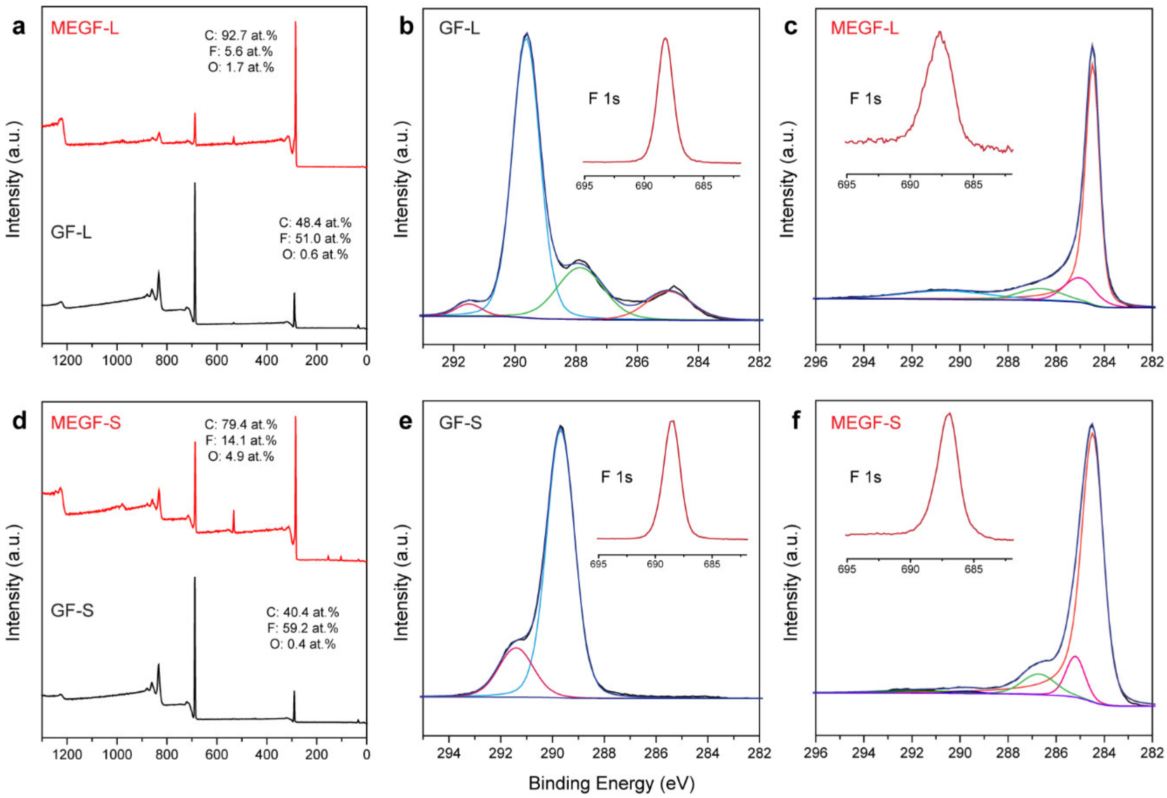

Figure S4. XPS surveys (**a**,**d**) and C 1s (**b**,**c**,**e**,**f**) and F 1s (insets in b, c, e, f) spectra of GF-L, MEGF-L, GF-S, and MEGF-S.

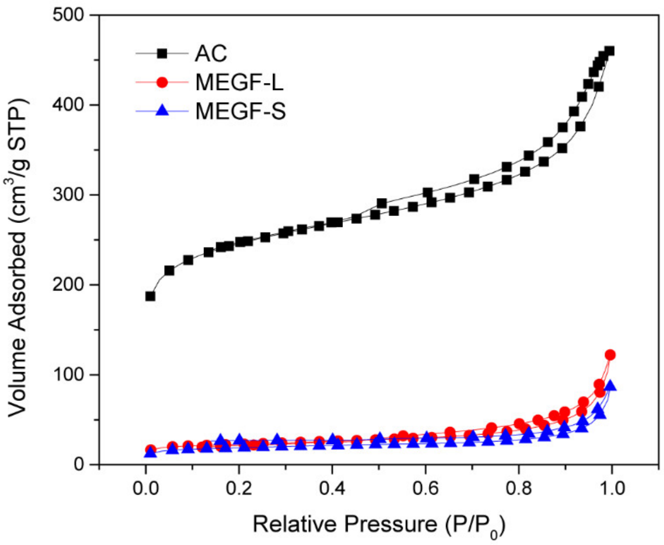

Figure S5. N_2 adsorption–desorption isotherms of activated carbon (AC), MEGF-L, and MEGF-S.
